# A Cross-Sectional Study Based on Forty Systematic Reviews of Foods with Function Claims (FFC) in Japan: Quality Assessment Using AMSTAR 2

**DOI:** 10.3390/nu15092047

**Published:** 2023-04-24

**Authors:** Hiroharu Kamioka, Hideki Origasa, Kiichiro Tsutani, Jun Kitayuguchi, Takahiro Yoshizaki, Mikiko Shimada, Yasuyo Wada, Hiromi Takano-Ohmuro

**Affiliations:** 1 Faculty of Regional Environment Science, Tokyo University of Agriculture, 1-1-1 Sakuragaoka, Setagaya-ku, Tokyo 156-8502, Japan; 2 The Institute of Statistical Mathematics, 10-3 Midori-cho, Tachikawa, Tokyo 190-8562, Japan; 3 The Institute of Seizon and Life Sciences, 4-5-1 Ginza, Chuo-ku, Tokyo 104-0061, Japan; 4 Physical Education and Medicine Research Center Unnan, 328 Uji, Unnan City 699-1105, Japan; 5 Faculty of Food and Nutritional Sciences, Toyo University, 1-1-1 Izumino, Itakura Town 374-0193, Japan; 6 Department of Nutrition, Faculty of Health Care, Kiryu University, 606-7 Asami, Midori City 379-2329, Japan; 7 Department of Health Promotion, National Institute of Public Health, 2-3-6 Minami, Wako City 351-0197, Japan; 8 Research Institute of Pharmaceutical Sciences, Musashino University, 1-1-20 Aramachi, Nishitokyo, Tokyo 202-8585, Japan

**Keywords:** food, supplement, randomized controlled trial, risk of bias, systematic review

## Abstract

Background: The Foods with Function Claims (FFC) was introduced in Japan in April 2015 to make more products available that are labeled with health functions. The products’ functionality of function claims must be explained by scientific evidence presented in systematic reviews (SRs), but the quality of recent SRs is unclear. This study assessed the quality of SRs in the FFC registered on the Consumer Affairs Agency (CAA) website in Japan. Methods: We searched the database from 1 April to 31 October 2022. Confidence in the methodological quality of each SR was evaluated by the AMSTAR 2 checklist. Results: Forty SRs were randomly extracted on the basis of the eligibility criteria and recruitment procedures. Overall confidence was rated as “high” (*N* = 0, 0%), “moderate” (*N* = 0, 0%), “low” (*N* = 2, 5%), or “critically low” (*N* = 38, 95%). The mean AMSTAR 2 score was 51.1% (SD 12.1%; range 19–73%). Among the 40 SRs, the number of critical domain deficiencies was 4 in 7.5% of SRs, 3 in 52.5% of SRs, 2 in 35% of SRs, and 1 in 5% of SRs. Registering the review’s protocol and comprehensive search strategies were particularly common deficiencies. Additionally, the risk of bias (RoB) was insufficiently considered. Conclusion: Overall, the methodological quality of the SRs based on the FFC, introduced eight years earlier, was very poor. This was especially true in the interpretation and discussion of critical domains, which had many deficiencies in terms of protocol registration, a comprehensive literature search strategy, and accounting for the RoB.

## 1. Introduction

In Japan, a new category of foods with health claims, the Foods with Function Claims (FFC) [1], was introduced in April 2015 in order to make more products available that were clearly labeled with certain health functions and to enable consumers to make more informed choices. The FFC allows manufacturers to submit their labeling to the Secretary-General of the Consumer Affairs Agency (CAA) in Japan, which indicates that the food is expected to have a specific functional effect on health. Although the CAA does not evaluate the safety and functionality of the submitted product, i.e., a notification system, the industry (applicant) must complete all the required procedures to submit a notification [2]. Additionally, all submitted information, including withdrawal and modification, is disclosed on the CAA website.

To substantiate the product’s functionality, the scientific evidence for the proposed function claims must be explained by one of two standard methods: clinical trials (CTs) such as randomized controlled trials (RCTs) or systematic reviews (SRs). The process for submitting a notification is published in the “Guidelines on Notification of Foods with Function Claims” (in Japanese) on the CAA website [2].

One problem with the FFC notification system, which is the adequacy of research reports, has been highlighted by the CAA and by research groups. The relevant CT must have been published in a peer-reviewed journal. However, the CAA examined 50 reported CTs in 2017 and determined that many had inappropriate protocols and methods for evaluating the risk of bias (RoB), and many had conflicts of interest (COI) [3]. One study identified problems with the reporting quality and the associated issues for 33 RCTs in the FFC; specifically, of the 29 check items in CONSORT 2010, the RTCs met only 13.8 items (47.6%) on average [4]. A 2021 study reported that compliance with the CT protocols in the FFC system was suboptimal in transparency. In addition to selective reporting, another problem was that the content of the intervention (test food) was intentionally concealed [5]. A subsequent 2022 study by these same researchers reported that randomization, deviations from the intended interventions, measurement of the outcomes, and selective reporting (particularly regarding the RoB, including a lack of ITT analysis, unknown compliance, and multiple outcome tests) seriously damaged the studies’ quality [6]. Thus, the clinical trials reported had many problems in their research methodology despite being published in academic journals.

On the other hand, more recent SR notifications submitted to the CAA have used a specific format based on the guidelines, and it was not required that the SR was published as an article in an academic journal. Because promoting deregulation is a national goal, SRs have the advantage of being easy to report for small- and medium-sized enterprises because they are less expensive than CTs such as RCTs. In fact, since the system’s launch in 2015, there have been 6247 cases reported as of 1 January 2023, of which 5906 (94.5%) have used an SR [7].

The CAA formatted the expert working group (methodologists for SRs) in 2016 in order to extract the issues for the appropriate operation of the FFC system and to perform verifications [8] according to the PRISMA guidelines [9]. In the SR notification, many items were omitted or explained poorly due to an insufficient understanding of the PRISMA checklist’s contents. A 2017 study by academic researchers [10] conducted a quality evaluation of SRs using the first edition of the “Assessment of Multiple Systematic Reviews” (AMSTAR) [11]. It was developed to assess the methodological quality of SRs, building upon previous tools, empirical evidence, and expert consensus.

A 2018 study by that same research group evaluated the quality of methodologies in SRs using the AMSTAR exactly as the previous academic study did [12]. Although two years had passed since the CAA’s report [8] and the academic reports [10,12], there were still very poor descriptions and/or implementation of the processes of study selection, data extraction, search strategy, evaluation of the methodology for the RoB, assessment of publication bias, and formulating conclusions based on methodological rigor and the scientific quality of the included CTs.

The need to improve the quality of SRs is not limited to food-related fields and is common in other healthcare research. A study that tracked the ratio of the number of SRs to the number of RCTs (SR/RCTs) that were published from 1995 to 2017 across various research fields found that this ratio approached 1.0 year by year [13]. In other words, the number of SRs and RCTs was almost the same, and many SRs were produced. Some researchers have suggested that the skyrocketing increase in SR production globally has led to overlaps, redundancies, and waste, as well as qualitative problems [14].

Regarding the assessment of multiple SRs, the original AMSTAR [11] is a well-known measurement tool, but quality reviewers were sometimes confused as to whether they should respond with “yes”, “no”, or “can’t answer” during their review process. Additionally, the reviewers seemed to have some ambiguity about the details of each item. AMSTAR 2 was developed to improve these issues in 2017 [15]. It has retained 10 of the original domains, has 16 items in total, has simpler response categories than the original AMSTAR, includes a more comprehensive user guide, and includes the identification of high-quality SRs. We need to use this new assessment tool to examine the quality of the SRs in the notification in order to properly maintain the FFC system and protect consumers. The purpose of this study was to use the AMSTAR 2 to assess the research quality of SRs reported as the scientific basis of functionality in the FFC system.

## 2. Methods

### 2.1. Eligibility and Exclusion Criteria (Target Articles)

**Figure 1:**
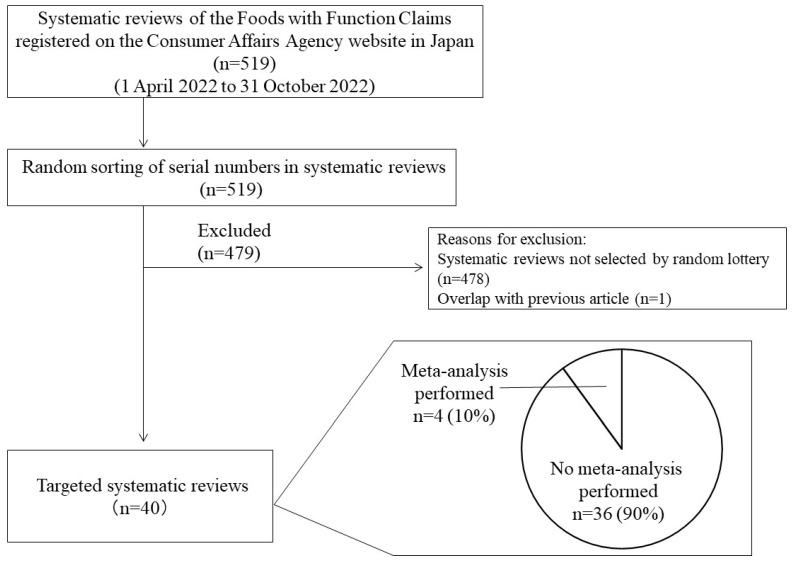
Flow of the trial process and study implementation.

All reported SR articles published on the CAA website during the year from 1 April 2022 to 31 October 2022 were reviewed. Initially, these included new and updated SRs belonging to the H series (identification number), specifically 519 articles (excluding retracted articles) classified as H1-H535 (Figure 1 and Table 1). In Japan, it is normal for both the government and companies to develop their businesses on an annual basis, so this period was set to collect the latest SRs for FY2022. Therefore, this study used SRs articles starting from the first in the H series. It was called the H series because FY2015, the year of its introduction, was the A series, and each subsequent year was identified in alphabetical order; thus, FY2022 was H or the eighth year.

From these, 40 articles were randomly extracted on the basis of the following eligibility criteria and recruitment procedures: (i) we excluded clinical and observational studies, (ii) we labeled the extracted SRs sequentially according to the notification number, (iii) we assigned random numbers to the labeled SRs in Microsoft Excel and selected the top 40 from the list after random sampling; (iv) if one SR had been notified in duplicate (e.g., for another product) even though it was an SR of the same function-related ingredient, only the first SR was selected to avoid double counting; in the event of a duplication, if the 41st SR was selected earlier, then it was still selected in the same manner.

### 2.2. Data Source

Target articles were downloaded from the CAA website [7].

### 2.3. Data Items and Evaluation of the Methodological Quality (AMSTAR 2)

AMSTAR 2 can cover SRs that include randomized and/or non-randomized studies of healthcare interventions. It consists of 16 check items, including 7 critical domains that can severely damage the quality of research.

Of the 40 articles, 36 (90%) were qualitative reviews (i.e., without a meta-analysis) and 4 (10%) performed a meta-analysis.

Four reviewing authors (JK, TY, MS, and YW) independently assessed the quality of the articles to ensure that variation was not caused by systematic errors during the study’s execution. Disagreements and uncertainties were resolved by discussion with another author (HK). Inter-rater reliability was calculated on a dichotomous scale using the percentage of agreement and Cohen’s kappa coefficient (*k*). Previously, the quality of studies was evaluated twice by four of the reviewer authors using the original AMSTAR. They underwent pre-consensus training using sample SRs with HK to increase the accuracy of the assessment utilizing the new tool, AMSTAR 2.

The percentage of descriptions present for all 16 of the check items for the quality assessment of articles was determined. Based on the percentage of the risk of poor methodology and/or bias, each item and the total score was assigned to one of the following categories: good description (80–100%), poor description (50–79%), or very poor description (0–49%). To compute the total percentage score, a score of 0 (answer “no”), 1 (answer “yes”), or 0.5 (answer “partial yes”) was assigned, then the scores were summed up and converted to a percentage scale. In the case of an SR in which no meta-analysis (MA) was undertaken, this percentage score included three non-applicable items. The denominators for such cases were therefore reduced accordingly in order to calculate a score based only on the remaining applicable items.

Furthermore, for each SR, the results of the evaluation per item were clarified, and an overall evaluation of reliability consisting of four stages was presented. The rating of the overall confidence in the results of the review was based on the following original criteria. The ratings were as follows: “high with no or one non-critical weakness”, where the systematic review provided an accurate and comprehensive summary of the results of the available studies that addressed the question of interest; “moderate with more than one non-critical weakness”, where the systematic review had more than one weakness but no critical flaws. It may provide an accurate summary of the results of the available studies that were included in the review; “low with one critical flaw with or without non-critical weaknesses” meant that the review had a critical flaw and may not provide an accurate and comprehensive summary of the available studies that addressed the question of interest. A study rated as critically low with more than one critical flaw with or without non-critical weaknesses meant that the review had more than one critical flaw and should not be relied upon to provide an accurate and comprehensive summary of the available studies. It should be noted that multiple non-critical weaknesses may diminish confidence in the review, and it may be appropriate to move an overall appraisal down from moderate to low confidence. However, in this study, no downgrading based on multiple non-critical weaknesses was performed. Critical items were only downgraded for reliability.

### 2.4. Evidence Table

As this study was not intended to provide the functionality of a functional component, a structured abstract (evidence table) was not produced.

### 2.5. Statistical Analysis

AMSTAR 2, unlike the original AMSTAR, was not designed to calculate the overall score, which, in this study, included 16 items. Therefore, only the frequency (as a percentage) of each item, the rating of overall confidence, and the percentage score (standard deviation: SD) for the 16 items were described. Statistical analysis was performed with SPSS version 23.0 (IBM Corporation, Armonk, NY, USA) for Windows.

### 2.6. Protocol Registration

The study methodology (protocol) was established on 19 November 2022. The study was registered as UMIN 000049558 by the University Hospital Medical Information Network Clinical Trials Registry (UMIN-CTR) in Japan (https://center6.umin.ac.jp/cgi-open-bin/ctr/ctr_view.cgi?recptno=R000056439) (accessed on 19 November 2022). UMIN-CTR is the largest CTR in Japan and joined the WHO registry network in October 2008. However, the UMIN-CTR cannot register the contents of all protocols in the input settings, so the complete protocol was stored in an online cloud, which can be viewed at this link: https://1drv.ms/b/s!AtViZRADbyukiG7zFy8oShYcWxRF?e=xgKqjL (accessed on 19 November 2022).

## 3. Results

### 3.1. Study Selection and Characteristics

The characteristics of all 40 articles are briefly summarized in Table 1. The articles were categorized according to their targeted food product: 25 (62.5%) were supplements, 14 (35.0%) were processed foods without supplements, and 1 (2.5%) was fresh foods. Of the 40 articles, 36 (90%) were qualitative reviews (i.e., without an MA) and 4 (10%) performed a MA.

For each SR, the outcomes were as follows: improvement of bowel movements (*n* = 7), inhibition of a rise in triglycerides after meals (*n* = 5), reduction of body fat (*n* = 5), suppression of postprandial increases in blood glucose (*n* = 3), relief of mental stress (*n* = 3), preservation of the skin’s elasticity (*n =* 3), improvement of cognitive function (*n =* 2), decreased blood pressure (*n =* 2), reduced fatigue (*n =* 2), improvements in decreased peripheral body temperature (*n =* 2), improvements in the contrast sensitivity of the eyes (*n =* 2), improvements in swelling (*n =* 1), improved vascular function (*n =* 1), improved knee joint function (*n =* 1), and improved immune function (*n =* 1).

### 3.2. Quality Assessment

We evaluated 16 items from the AMSTAR 2 checklist in more detail (Table S1). The individual quality evaluation results were sorted so that the name of the company that submitted the notification was not specified. The inter-rater reliability metrics for the quality assessment indicated substantial agreement (91.9%, *k* = 0.849) for all 608 items (16 items multiplied by 38 SRs). The two SRs that were used during pre-consensus training were not included in the calculation of internal validity.

**Figure 2:**
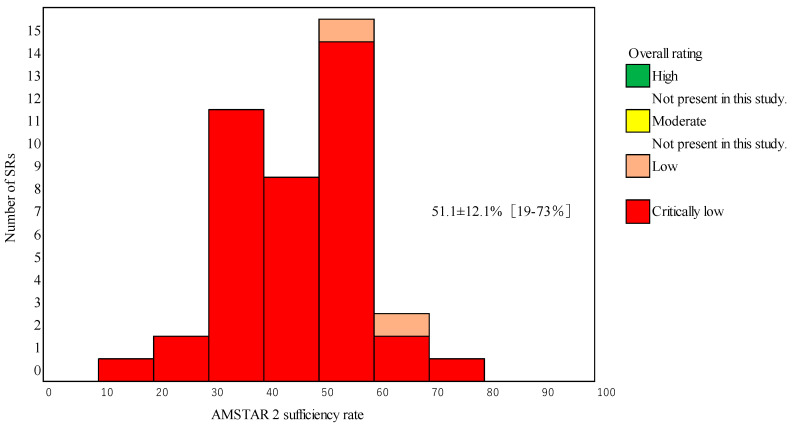
Histogram of the number of SRs in the respective AMSTAR 2 percentage scores and color-coded ratings of overall confidence in the results of the SRs. Mean ± SD [Range]. The overall confidence ratings assigned to the 40 articles were “high” (*N* = 0, 0%), “moderate” (*N* = 0, 0%), “low” (*N* = 2, 5%), and “critically low” (*N* = 38, 95%). The mean AMSTAR 2 percentage score was 51.1% (SD 12.1%), with a wide range of 19% to 73%.

The overall confidence ratings assigned to the 40 articles were “high” (*N* = 0, 0%), “moderate” (*N* = 0, 0%), “low” (*N* = 2, 5%), and “critically low” (*N* = 38, 95%) (Figure 2). The mean AMSTAR 2 percentage score was 51.1% (SD 12.1%), with a wide range from 19% to 73%.

#### 3.2.1. Critical Domains

**Figure 3:**
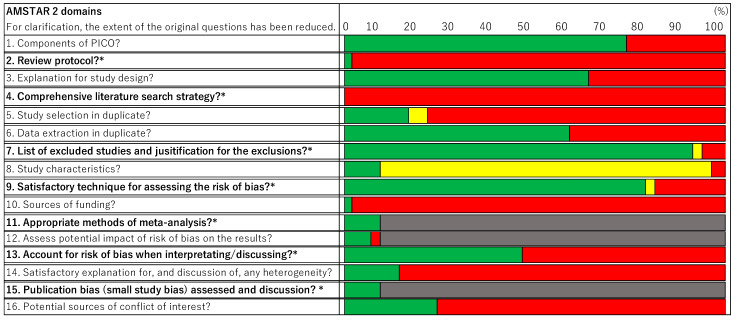
Methodological quality of the 40 SRs according to the 16 items of AMSTAR 2. Yes, green; Partly yes, yellow; no, red; no meta-analysis conducted, grey. * Critical domains. The critical domains were very poorly described and/or implemented (0–49%): “#2 Review protocol?” (*N* = 2, 5%), “#4 Comprehensive literature search strategy?” (*N* = 0, 0%), and “#13 Accounting for the risk of bias when interpreting/discussing the results?” (*N* = 19, 47.5%).

Seven critical domains had good descriptions and/or implementation (80–100%) for the following items (Figure 3): “#11 If a meta-analysis was performed, did the review authors use appropriate methods for statistical combination of the results?” (*N* = 4, 100%), “#15 If they performed a quantitative synthesis, did the review’s authors carry out an adequate investigation of publication bias (small study bias) and discuss its likely impact on the results of the review?” (*N* = 4, 100%), “#7 Did the review’s authors provide a list of excluded studies and justify the exclusions?” (*N* = 37, 92.5%), and “#9 Did the review’s authors use a satisfactory technique for assessing the risk of bias (RoB) in individual studies that were included in the review?” (*N* = 32, 80%).

The other items were very poorly described and/or implemented (0–49%): “#2 Did the report of the review contain an explicit statement that the review methods were established prior to the conduct of the review and did the report justify any significant deviations from the protocol?” (*N* = 2, 5%), “#4 Did the review’s authors use a comprehensive literature search strategy?” (*N* = 0, 0%), and “#13 Did the review’s authors account for RoB in primary studies when interpreting/discussing the results of the review?” (*N* = 19, 47.5%). Sufficiency in the literature search strategy for critical domains included (i) a small number of databases in English and only MEDLINE/PubMed and one other database (DB), (ii) no description of how language restrictions were made, and (iii) a literature search using only the DB above, and no other search engines, manual searches, citation searches, referrals, etc.

#### 3.2.2. Non-Critical Domains/Weaknesses

For the other (non-critical) domains, there were poor descriptions and/or implementation (Figure 3): “#1 Did the research questions and inclusion criteria for the review include the components of PICO?” (*N* = 31, 77.5%), “#3 Did the review’s authors explain their selection of the study designs for inclusion in the review?” (*N* = 26, 65%), “#6 Did the review’s authors perform data extraction in duplicate?” (*N* = 24, 60%), and “#12 If a meta-analysis was performed, did the review’s authors assess the potential impact of the RoB in individual studies on the results of the meta-analysis or other evidence synthesis?” (*N* = 3, 75%).

Other non-critical items also had very poor descriptions and/or implementation: “#5 Did the review’s authors perform study selection in duplicate?” (*N* = 7, 17.5%), “#8 Did the review’s authors describe the included studies in adequate detail?” (*N* = 4, 10%), “#10 Did the review’s authors report on the sources of funding for the studies included in the review?” (*N* = 1, 2.5%), “#14 Did the review’s authors provide a satisfactory explanation for, and discussion of, any heterogeneity observed in the results of the review?” (*N* = 6, 15%), and “#16 Did the review’s authors report any potential sources of conflict of interest, including any funding they received for conducting the review?” (*N* = 7, 17.5%).

#### 3.2.3. Common Critical Domains That Caused Degradation

**Figure 4:**
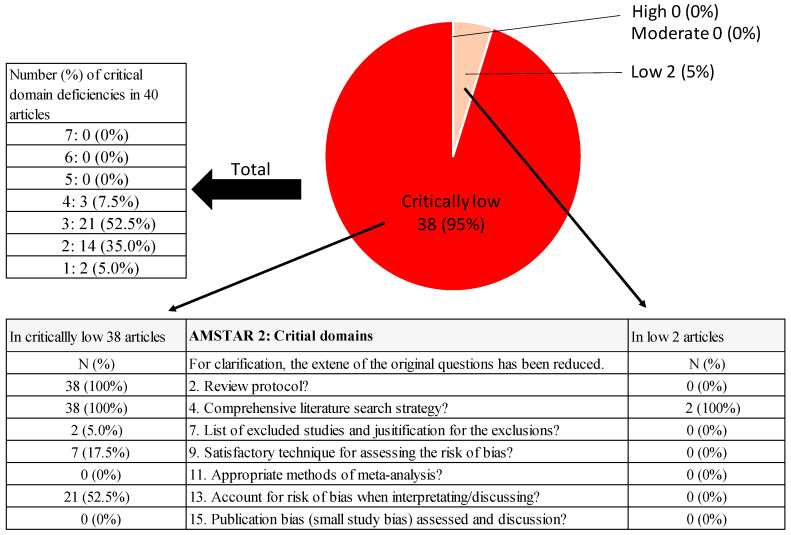
Common critical domain deficiencies by overall confidence.

Several common critical domains caused a degradation of the overall confidence (Figure 4). Among the 40 SRs, the number of deficiencies in the critical domains was 4 in 7.5% of the SRs, 3 in 52.5% of the SRs, 2 in 35% of SRs, and 1 in 5% of the SRs. The registration of a review protocol and comprehensive search strategies were particularly common deficiencies. In addition, there was insufficient consideration of the RoB.

## 4. Discussion

This was the first study to assess the research quality of SRs reported as the scientific basis of functionality in the Japan FFC system by using the AMSTAR 2 tool. In the notification system without examinations, we were able to clarify the limits on SRs that were produced by the food industry in Japan. Unfortunately, most of the reviewed SRs had critically low reliability, a finding that should trigger a more rigorous evaluation of the rapidly increasing number of SRs on foods. We propose that this study will be helpful to researchers and government officials who want to introduce new health claims in advanced countries.

### 4.1. Main Findings

The fact that many deficiencies have been observed in articles published during the eight years since the introduction of the FFC system indicates that notifications have increased without a sufficient understanding of the research methodology of SRs, e.g., planning, implementation, and reporting. In 2017 [10] and 2019 [12], studies that evaluated the quality of SRs in the FFC system, which used the first edition of AMSTAR, found many poor SRs, but no trend of improvement trend was observed.

In particular, the fact that the critical domain of the review’s protocol was not registered or its whereabouts were not clear was common and caused significant damage to the quality of the research. If the protocol is not clear, doubts such as PICO replacement arise, as represented by selective outcomes, and the credibility of an SR is certainly lessened. Under this system, SR notifications by companies are linked to sales strategies, so competitors constantly monitor SR notifications. As a result, companies may fear that their competitors will know about the new products and functional ingredients they plan to sell in the future. This system, made possible by Japan’s deregulation policy, seems to have a complicated background that directly involves businesses. In fact, under the current guidelines [2], notification of SRs is required in accordance with the PRISMA checklist, but pre-registration of the protocol is not mandatory.

Thus, many problems arise with the quality of SRs. An SR is a notification system, and there is no evaluation by the CAA, so low-quality reports are mass-produced. One improvement would be to require that only SRs published in peer-reviewed journals be used for notification. Currently, another type of notification would require a single clinical trial article to be published in a peer-reviewed journal. An SR would meet the same standards if it had to be published in an academic journal.

### 4.2. Findings in Context

Our findings of the poor methodological quality of SRs in the food field are in line with those from assessments of the methodological quality of SRs that used the AMSTAR 2 tool in other fields, including acupuncture [16,17], dentistry [18], Tai Chi [19,20], surgery [21,22], major depression in adults [23], chronic obstructive pulmonary disease [24], cardiovascular risk [25], physical activity [26], pancreatic cancer [27], chronic heart failure [28], cancer-related fatigue [29], Alzheimer’s disease [30], chronic spinal pain [31], neonatal pain [32], hip fractures [33], and atopic eczema [34]. Although the target patients or participants and interventions in those studies differed between each study, all demonstrated that the majority of SRs included had low methodological quality (“low” or “critically low”). In contrast, some research areas, such as COVID-19 [35], have recently produced many high-quality SRs.

A lack of protocol registration was the most problematic issue with the articles included in this study, and this was quite common in previous studies in other research fields [18,23,24,25,28,30,34]. PROSPERO [36] is a well-known register of SRs, and future SR implementers must be registered. In Japan, many researchers use UMIN-CTR when conducting a CT, and SR registration is also possible. However, one inconvenience of UMIN-CTR is that it is not possible to provide a sufficient amount of text on this site. It is, therefore, sometimes necessary to post a link and store it semi-permanently in the cloud or some other data storage location where anyone around the globe can access it.

Many insufficient literature searches have been conducted. In recent years, multiple new information media sites have arisen, and it is necessary to detail their inclusion in search strategies in order not to overlook a CT that met the eligibility criteria, as well as to avoid publication bias. The importance of such a search strategy is demonstrated in the latest PRISMA 2020 guidelines [37], and the linked PRISMA-S (in advanced countries) explains the strategy in detail [38]. Advance planning according to these checklists will certainly improve the quality of SRs.

Consideration of the method of evaluating the RoB and how it was implemented had a negative impact on the quality of SRs in some previous studies [17,18,23,24,35]. Many of the SRs in this study were evaluated using an older version of the Cochrane Collaboration’s tool for assessing RoB in randomized trials [39]. Although it is important to use the latest evaluation tools based on specific signaling questions and algorithms such as RoB2 [40], the essence is to explain whether the interpretation of the RoB in the highly biased CTs was excluded and how it was reflected in the conclusion.

### 4.3. Relevance to the GRADE Approach (Grading of Recommendations Assessment, Development, and Evaluation)

The GRADE approach is a prominent method of assessing the certainty in evidence and the strength of the recommendations in healthcare [41]. It is often used for the formulation of clinical practice guidelines and is evaluated based on five items: RoB, imprecision, inconsistency, indirectness, and publication bias. Since the purpose of the FFC system is to promote health rather than cure or prevent disease, P in the acronym PICO is defined as a healthy adult, and an SR is not allowed to include sick people. Since GRADE evaluations are often used for SRs of CTs that were originally designed to measure the therapeutic effect on a certain disease, it was not adopted as an evaluation tool in this study.

However, there may be considerable correlations between the certainty of the results of the evidence and the results of quality assessments based on AMSTAR 2. Recently, many umbrella reviews have been performed that used both AMSTAR 2 and GRADE in healthcare settings [22,25,27,32,33,34]. These studies were consistently determined to be “low” and “very low” according to the GRADE evaluation when the ratings were also “low” or “critically low” in AMSTAR 2. A future study in the nutrition field is expected to use a research methodology that will rigorously investigate the correlation between these two methods.

### 4.4. Challenges for Improving the Quality of SRs in the FFC

Considering the pivotal role that SRs have in medical decision making due to their transparent, objective, and replicable processes, a failure to appreciate and regulate the problems with these highly cited research designs is a threat to credible science [42]. Even under the FFC system, it will not be acceptable to postpone improvements in the quality of SRs.

Here, we summarized the challenges for improving the quality of SRs in the FFC (Table 2), which are extremely important points to be considered by companies and the food industry, academia, and the CAA of Japan. Companies and the food industry should conduct research based on the guidelines of AMSTAR 2, PRISMA 2020 [37], PRISMA-S [38], PRISMA-P [43], and GRADE [41]. If the method used to perform an SR is unknown, it may be necessary to obtain guidance from experts. Academic researchers should conduct regular surveys, clearly explain the results to regulators, society, and consumers (using plain language), and present proposals for improvement. Regulatory guidelines should oblige the authorities to require companies to pre-register the protocol and should also encourage companies to give notification of an SR that is published in an academic journal in order to guarantee its quality. The active involvement of all parties to achieve that objective would be essential.

## 5. Limitations

This review had several limitations that should be acknowledged. First, publication bias was possible. Although we performed random sampling, the deviation from all SRs as a population was unknown. Second, although we placed importance on the descriptions being present for each item in the SRs, we did not clarify unclear points with the authors. Third, our study design focused on the quality of SRs; therefore, we could not validly assess the functional mechanism of any product reviewed in the SRs. Additionally, because we did not conduct a retrospective analysis of the quality of the primary studies cited or used as references that were described in the SRs, the functionality of functional substances or finished products could not be addressed. Fourth, this study did not use the ROBIS tool, which can assess the RoB in SRs with higher sensitivity, and we believe that it could also be used to better clarify the overall improvement of SRs. Zeraatkar et al. [44] evaluated 140 reviews in the field of nutrition using the ROBIS and reported the effectiveness of their RoB assessments. Sixth, we were unable to make comparisons with the GRADE evaluation, which indicates the certainty of the evidence. Lastly, we were unable to link individual results of quality evaluations with company names (product names and functional substances) because of the potential risk of civil suits and other serious problems.

## 6. Conclusions

Overall, the methodological quality of SRs based on the FFC, even eight years after the introduction of the FFC, was very poor. This was especially true in the interpretation and discussion of critical domains, which had many deficiencies in the areas of protocol registration, comprehensive literature search strategies, and accounting for the RoB. These deficiencies suggest that methodological education and enlightenment are necessary to improve the quality of SRs in newly notified or updated SRs in the future. Considering the specific nature of SRs for the food business, such guidelines should include pre-registration of the protocol and a recommendation to publish the SR in an academic journal before notification in order to have an appropriate SR methodology established.

## Figures and Tables

**Table 1:** Characteristics of systematic reviews of the Foods with Function Claims.

No.	Product Name	Food Business Operator	Classification of Food 1 Supplement 2 Processed Food 3 Fresh Produce	Functional Substance
H488	*Kokunaisan Organic Kikuimo Tea*	Aim Co., Ltd.	2	Inulin
H464	*Nisshin MCT Mayonnaise Sauce*	The Nisshin OilliO Group, Ltd.	2	Medium chain triglyceride (octanoic acid, decanoic acid)
H339	*BIKOKUSAI chinese soup*	BROOK’S Co., Ltd.	2	Indigestible dextrin (dietary fiber)
H24	*Pilkul miracle care*	Nissin York Co., Ltd.	2	Lactic acid bacterium NY1301 strain
H1	*Terminalia slim +B*	Aminocells Corp.	1	Gallic acid made from *Terminalia berylica*
H39	*Hapicolla Stick*	Nitta Gelatin Inc.	2	Low-molecular-weight collagen peptide made from fish
H173	DHA900	Nakahara Co., Ltd.	1	DHA⋅EPA
H381	*Kekkan shinayaka elastin supplement*	HAKUJU INSTITUTE FOR HEALTH SCIENCE Co., Ltd	1	Elastin peptide made from bonito
H414	*im Protein Resilie*	Ortho corporation	1	Salmon nasal proteoglycan
H195	*Hisamitsu^®^ metasapo^®^*	HISAMITSU PHARMACEUTICAL CO.,INC.	1	Tea catechin, Epigallocatechin gallate (EGCg)
H337	*Swellieve*	DIANA Co., Ltd.	1	Genquanine 5-O-β-primeveroside, Mangiferin, Piperines made from *hihatsu*
H236	*Psyllium fiber 100%*	YAMAMOTO KANPOH PHARMACEUTICAL CO., LTD.	1	Dietary fiber made from *psyllium seed coat*
H318	*Shibo Chuiho EXb*	MG PHARMA, Inc.	1	Valine-Valine-Tyrosine-Proline made from *globin*
H245	*YUTAKANI*	SONOKO Co., Ltd.	1	Camellia saponin B2
H15	Rrefreshing life with lactobacillus	Willumina, Inc.	1	Bacillus coagulans SANK70258
H438	No response was received from the company.	Medione, Inc.	1	Elastin peptide made from bonito
H357	*Shokuji To Salacia*	Asahi Bussan Co., Ltd.	1	Salacinol made from *Salacia*
H290	MINUTE MAID PLASMA LACTO. IMMUNITY CARE	Coca-Cola (Japan) Company, Limited	2	L. lactis strain plasma
H120	*PURU-Kira Mamoru-kun*	Nippi, Incorporated	1	Collagen peptide made from fish
H526	*Du-Zhong Tea*	Kobayashi Pharmaceutical Co., Ltd.	2	Geniposidic acid
H358	*Umakara Kimuchi*	PICKLES CORPORATION	2	Lb. plantarum PIC-NBN22, Fructo-oligosaccharide
H301	*Rosemary a*	S&B FOODS, Inc.	1	Rosmarinic acid made from *rosemary*
H527	*CUTTO MAINTE*	KARADA NI EIYO, Inc.	1	Piperines made from *hihatsu*
H53	*mimamoru a*	Ogaland Co., Ltd.	1	Lutein
H456	The product has not been named in English yet.	Pyuru Co., Ltd.	1	Lutein, Zeaxanthin
H91	*Nagarurumo*	Ortho Corporation	1	Piperines made from *hihatsu,* Bacillus coagulans SANK70258
H465	Production and sales were discontinued.	YAMASA CORPORATION	2	Inulin
H155	*Eyebon supple a*	Kobayashi Pharmaceutical Co., Ltd.	1	Lutein, Zeaxanthin
H149	*Mirasoup consomme flavor*	PremiumCosme Inc.	2	Isoflavones made from *kudzu* (Tectorigenins), Glucosylceramide made from rice
H415	*SLIM BLOCK CHOKATSU PRO*	Nihon Yakken Co., Ltd.	1	Gallic acid made from *Terminalia berylica*, Bacillus coagulans SANK70258
H444	*Theracurmin 3E*	Theravalues Corporation	1	Curcumin
H367	*Cirneco Rosso*	Croix Co., Ltd.	2	GABA
H455	*SUYASUYANOTANE+*	Facelabo Co., Ltd.	1	GABA
H338	The product has not been named in English yet.	Mikakuto Co., Ltd.	1	Plant lactobacillus K-1(L. casei 327)
H126	Green juice for those worried about cholesterol or triglyceride	Taisho Pharmaceutical Co., Ltd.	2	Ellagic acid
H141	*”KARADA-ni” Euglena Muscat & Herbal Flavor*	Euglena Co., Ltd.	2	Paramylon made from *Euglena Gracilis* (β-1,3-glucan)
H29	*Joshu chicken (breast meat)*	KURICHIKU, Inc.	3	Imidazole dipeptide
H428	*Slim apple*	Setagaya Natural Foods Co., Ltd.	1	Procyanidins made from apple
H19	*Terminalia slim +A*	Aminocells Corp.	1	Gallic acid made from *Terminalia berylica*
H360	*Oligosmart 100 milk chocolate*	Meiji Co., Ltd.	2	Fructooligosaccharides

The articles were categorized according to their targeted food product: 25 (62.5%) were supplements, 14 (35.0%) were processed foods without supplements, and 1 (2.5%) was fresh foods.

**Table 2:** Challenges for improving the quality of SRs in the Foods with Function Claims.

For Companies or Food Industry	
#1	The applicants should conduct research based on AMSTAR 2, PRISMA 2020, PRISMA-S, PRISMA-P, and GRADE.
#2	The applicants should receive guidance from searchers, biostatistics experts, and research methodologists on literature search methods, meta-analysis methods, and bias assessments, respectively.
**For academia**	
#3	Academia researchers should conduct regular surveys and make proposals for improvement since the authorities in charge cannot evaluate the quality of individual SRs because of the notification system.
**For the Consumer Affairs Agency in Japan**	
#4	The authority should be obliged in the guidelines to require companies to pre-register the protocol.
#5	The authority should encourage companies to notify them if an SR is published as an article in an academic journal.

## Supplementary Materials

The following supporting information can be downloaded at: https://www.mdpi.com/article/10.3390/nu15092047/s1. Table S1: Results of the evaluation using AMSTAR 2.

## Abbreviations

CAA: Consumer Affairs Agency; CT: clinical trial; FFC: Foods with Function Claims; MA: meta-analysis; RCT: randomized controlled trial; RoB: risk of bias; SR: systematic review.
